# Contact area and pressure changes of patellofemoral joint during stair ascent and stair descent

**DOI:** 10.1186/s12891-023-06882-0

**Published:** 2023-09-28

**Authors:** Xiaomeng Wang, Huixin Liu, Zhenyue Dong, Xiaobo Chen, Chenyue Xu, Gang Ji, Huijun Kang, Fei Wang

**Affiliations:** 1 Foot and Ankle Surgery, The Third Hospital of Hebei Medical University, Shijiazhuang, China; 2 Ultrasound medicine department, The Third Hospital of Hebei Medical University, Shijiazhuang, China; 3 Department of Joint Surgery, The Third Hospital of Hebei Medical University, Shijiazhuang, China

**Keywords:** Patellofemoral joint, Pressure, Contact area, Stair ascent, Stair descent, Finite element modelling

## Abstract

**Purpose:**

To investigate the differences of patellofemoral joint pressure and contact area between the process of stair ascent and stair descent.

**Methods:**

The finite element models of 9 volunteers without disorders of knee (9 males) to estimate patellar cartilage pressure during the stair ascent and the stair descent. Simulations took into account cartilage morphology from magnetic resonance imaging, joint posture from weight-bearing magnetic resonance imaging, and ligament model. The three-dimension models of the patella, femur and tibia were developed with the medical image processing software, Mimics 11.1. The ligament was established by truss element of the non-linear FE solver. The equivalent gravity direction (-z direction) load was applied to the whole end of femur (femoral head) according to the body weight of the volunteers, and the force of patella was observed. A paired-samples *t*-test or *Wilcoxon* rank sum test to make comparisons between stair ascent and stair descent. Statistical analyses were performed using *SPSS* 22.0 using a *P* value of 0.05 to indicate significance.

**Results:**

During the stair descent (knee flexion at 30°), the contact pressure of the patella was 2.59 ± 0.06Mpa. The contact pressure of femoral trochlea cartilage was 2.57 ± 0.06Mpa. During the stair ascent (knee flexion at 60°), the contact pressure with patellar cartilage was 2.82 ± 0.08Mpa. The contact pressure of the femoral trochlea cartilage was 3.03 ± 0.11Mpa. The contact area between patellar cartilage and femoral trochlea cartilage was 249.27 ± 1.35mm^2^ during the stair descent, which was less than 434.32 ± 1.70mm^2^ during the stair ascent. The area of high pressure was located in the lateral area of patella during stair descent and the area of high pressure was scattered during stair ascent.

**Conclusion:**

There are small change in the cartilage contact pressure between stair ascent and stair descent, indicating that the joint adjusts the contact pressure by increasing the contact area.

**Supplementary Information:**

The online version contains supplementary material available at 10.1186/s12891-023-06882-0.

## Introduction

The principal functions of patella are to increase the moment arm of the quadriceps mechanisms and to transmit the tensile forces of quadriceps muscle to the patellar tendon. The stair ascent and stair descent are common activities of daily life that is biomechanically and physiologically more challenging than level walking. Analysis of the biomechanical and pathomechanics of knee requirements during the stair ascent and stair descent can add to our understanding of the diverse demands of this common activity in human motion. Comparing with walking, only a small number of studies have investigated stair ascent and stair descent of normal person [1–4].

The distribution of forces across the patellar articular surface during knee flexion involves the complex and dynamic interplay between soft tissue restraints and the bony geometry, so determination of the pressure distribution in vivo studies remains a challenging [5–7]. In order to solve this problem, the computational models of patellofemoral joint mechanics have been developed to understand patellofemoral function [8–10]. In particular, the finite element (FE) method offers the ability to predict spatial and temporal variations in stress, strain, and contact area/forces [11, 12]. Therefore, the finite element method is used to study the biomechanical characteristics of patellofemoral joint.

Earlier in vitro studies have demonstrated that, in weight bearing, contact pressures within the patellofemoral joint increase as the knee flexes from 0° to 90° [13, 14]. Comparison to descent stair, larger ranges of knee flexion angle and knee flexion moment are required during ascent stair. Reilly and Martens identified that the patellofemoral (PF)joint reaction force can be 0.8 times body weight during level walking [15]. The patellofemoral joint pressure was three times higher when climbing stairs than when walking horizontally [16]. The person extends the leg to the next step and contacts the stair at a higher flexion angle during ascent stair than that during stair descent [15, 17]. So, the patellofemoral joint contact area and pressure varies with the knee flexion angle in daily activities.

The goal of this study was to investigate the contact area and pressure changes of the patellofemoral joint during stair ascent and stair descent. It was assumed that the contact area and pressure of patellofemoral joint on the stair ascend than that on the stair descend.

## Materials and methods

### Subjects.

Experimental data were collected from 9 volunteers (9 males) without knee disorders or other neuromuscular disease that could affect the results of the experiment, and history of previous surgery. For experimental precision, these volunteers with height of 175 to 185 cm and weight of 70 to 80 kg were selected. Basic information was summarized in Table 1. For the accuracy of the experimental data, the gender, height and weight of the enrolled volunteers were restricted. Previous work estimating weightbearing patellofemoral joint contact area showed that males have 34% greater contact area compared with females [6]. All participants provided written informed consent and the experimental protocol was approved by the Ethics Committee of the Third Hospital of Hebei Medical University.

**Table 1:** Demographic characteristics of the participants

Basic Information	Numerical Value
Age (y)	24.8 ± 1.56
Height (m)	1.8 ± 0.02
Body mass (kg)	77.3 ± 3.87
BMI (kg/m^2^)	23.9 ± 0.88

**Fig. 1:**
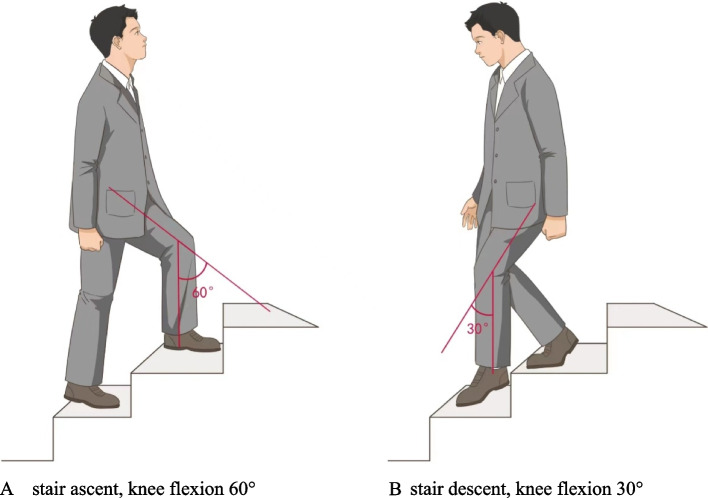
Schematic diagrams of stair ascent and stair descent. **A** stair ascent, knee flexion 60. **B** stair descent, knee flexion 30°

Two instantaneous actions were selected during the ascent stair and descent stair (knee flexion 60° on the stair ascent and 30° on the stair descent). (Fig. 1)There postures were chosen because they are positions at which peak knee extension moments are produced during stair ascent and stair descent [15].

**Fig. 2:**
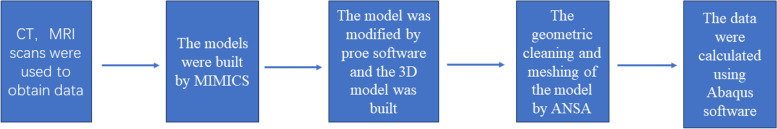
A flow chart of finite element analysis

The flow chart of obtaining experimental data and establishing the model was shown in Fig. 2. The geometrical data were obtained from computed tomography (CT) and magnetic resonance imaging (MRI) image acquisition from these healthy young male volunteers. Thin-slice CT (Spiral CT: Siemens 64-slice spiral CT, scan thickness 0.625 mm) was performed with 60° of knee flexion (upstairs) and 30° of knee flexion (downstairs), respectively. To segment the geometry of cartilage and the ligament of the knee, MR images of the knee was acquired with 3.0-T Siemens MR scanning system (Verio, Siemens, Erlangen, Germany) using fat-suppressed TIRM sequence (repetition time, 3500 ms; echo time, 32 ms; flip angle, 120°; matrix,320 × 224; field of view, 16 × 16 cm; slice thickness, 5 mm; scan time, 00:42 min). A 25% body weight resistance was provided through the pulley system to the footplate. Sagittal plane images were obtained at 30° and 60° of knee flexion.

### Strategies for finite element models

**Fig. 3:**
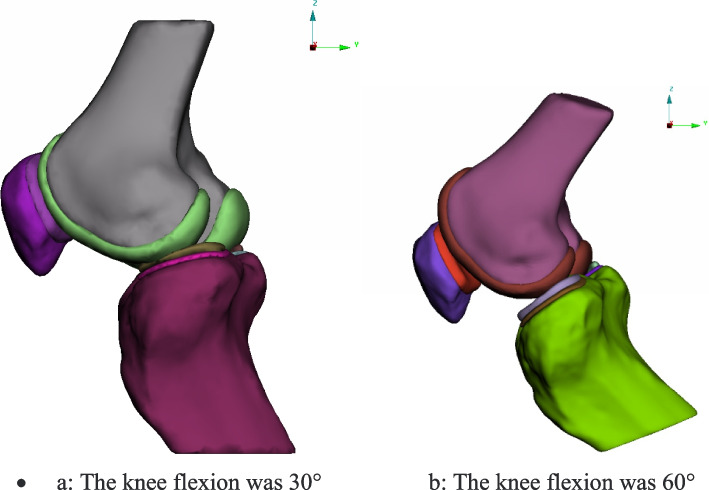
The 3D model and global coordinate system established at knee flexion at 30° and 60°. **a**: The knee flexion was 30° **b**: The knee flexion was 60°

The three-dimension models of the patella, femur and tibia were developed with the medical image processing software, Mimics 11.1 (Materialise, Inc., Belgium). The three-dimension models were shown in Fig. 3.

The CT and MRI scan data were transferred into the computer and the geometric model of the bone (femur, patella and tibia) the cartilage and meniscus were constructed by the Mimics11.1 software (Materialise, Inc., Belgium). Then the model was input into Pro/Engineer 4.0 software, and the advanced surface was established, meanwhile the geometry was corrected for establishing a complete knee joint model. To create a more perfect model, anatomical features were combined with digital models. The specific parameters are shown in Table 2.

**Table 2:** Setup Materials

	Modulus of elasticity (MPa)	Poisson's ratio
Cortical bone	12000	0.3
Cancellous bone	100	0.2
Meniscus	59	0.475
Cartilage	15	0.3
Ligament	48	0.3

**Fig. 4:**
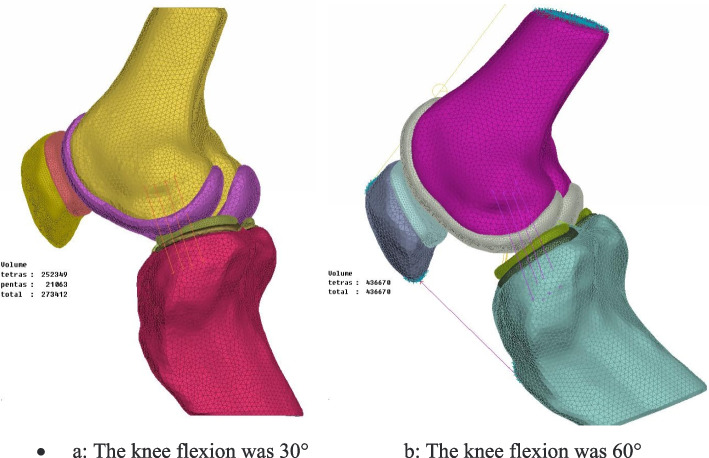
The mesh models of knee with 30° and 60° flexion were established respectively by the ANSA software. **a**: The knee flexion was 30° **b**: The knee flexion was 60°

The Meniscus was modeled with an elastic modulus of 59 MPa and a Poisson ratio of 0.475 [18, 19].The cartilage was modeled using eight-noded linear elastic solid elements with an elastic modulus of 15 MPa and a Poisson ratio of 0.3 [19, 20]. The cortical bone was modeled with an elastic modulus of 12,000 MPa and a Poisson ratio of 0.3 [21]. The Cancellous bone was modeled with an elastic modulus of 100 MPa and a Poisson ratio of 0.2 [21]. This linear elastic model is a reasonable assumption for dynamic activities such as a stair climb task. The patella ligament and quadriceps tendon were modeled using one-dimensional tension-only connector elements. The patella ligament was modeled distributed evenly across the attachment areas of the tibia and femur. The mesh models of knee with 30° and 60° flexion were established respectively by the ANSA software, utilizing the subdivided tetrahedral elements, truss elements (ligaments) and the data structure to fast search. The bulk element and the truss element of ligament are locally encrypted to ensure computational accuracy and speed. The tetrahedral elements were adopted C3D4 element and T3D2 element. As shown in Fig. 4

The ligament was established by truss element of the non-linear FE solver (ABAQUS Explicit, SIMULIA, Providence, RI). The nonlinear truss element was only strained but not compressed, which was consistent with the characteristics of knee ligament. Considering the lubrication and frictional force in the joint, a friction coefficient was modeled as 0.02 using surface-to-surface contact pairs.

**Fig. 5:**
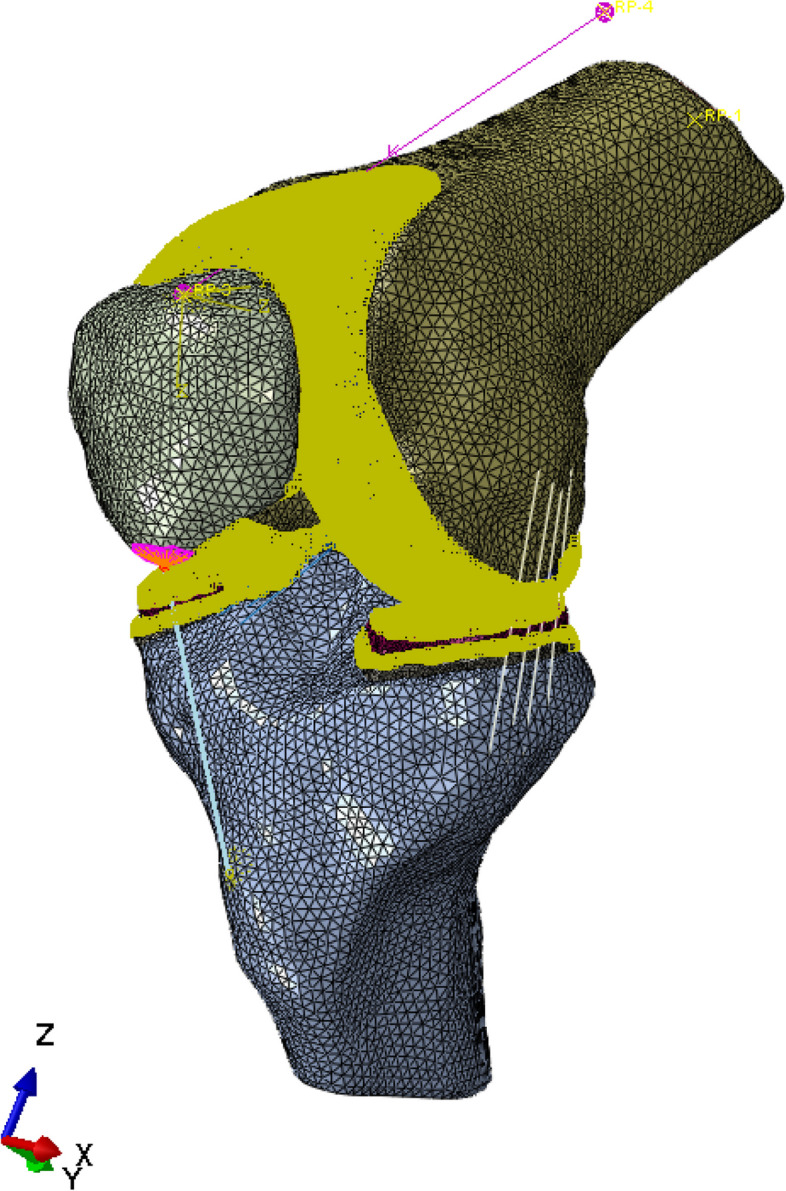
Schematic representation of tendon-bone coupling constraints

In order to facilitate loading and reduce loading stress concentration in the model, reference points were established in the connection area between suprapatellar muscle and patella, the infrapatellar ligament and patella, and the infrapatellar ligament and tibia. The reference was shown as Fig. 5. According to the computational characteristics of the model, cortical bone, cancellous bone and cartilage were constrained by common nodes. The quadriceps femoris was simulated with a spring, which was used to simulate the interaction between the muscle and the bone.

### Joint loading and muscle force model

Considering the gait of ascending and descending stair, this simulation only targeted a certain quasi-static angle in the ascending and descending process, so fixed constraints were applied to the bottom of the tibia. On account of the stair ascent and descent were carried by one leg, the equivalent gravity direction (-z direction) load was applied to the whole end of femur (femoral head) according to the body weight of the volunteers, and the force of patella was observed.

**Fig. 6:**
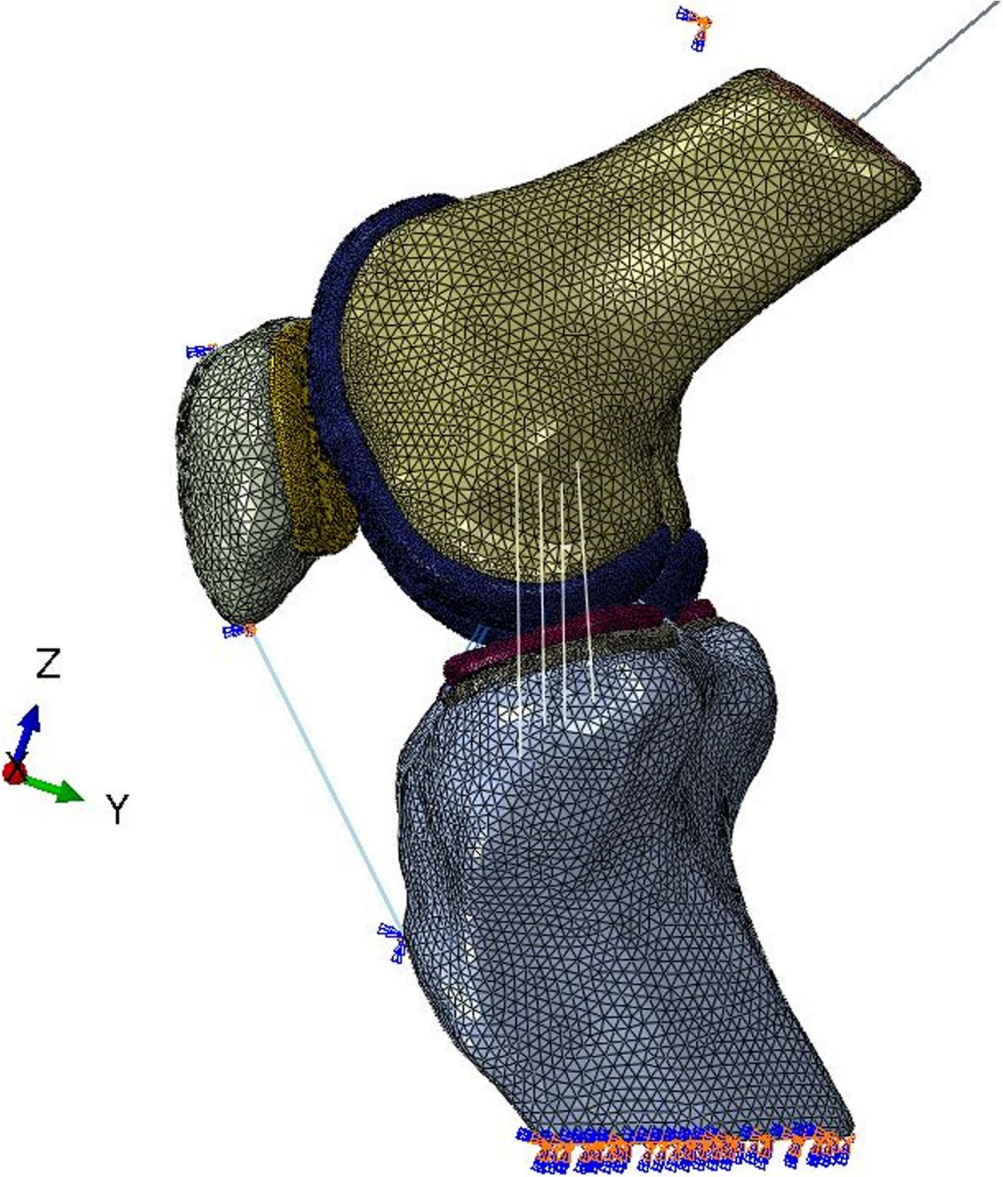
Simulation of gravity

The length from the knee joint to the femoral head was measured by X-ray of the full length of the lower limb. Considering the torque caused by the length of femur and the position of the center of gravity. An equivalent gravity direction (-Z direction) load is applied to the entire femur end (femoral head) according to the body weight of the volunteer. The BEAM extension was used to determine the center of gravity position after measurement, and simulation was performed. (Fig. 6).

### Data analysis and statistical methods

Statistical analyses were performed using *SPSS* 22.0 (IBM Co, Armonk, New York, USA) using a *P* value of 0.05 to indicate significance. The effects of variations were quantified in magnitude of the force and contact area of PF. Assessment of the pressure of patellofemoral cartilage focused on the maximum pressure of patellofemoral cartilage. Assessment of the contact pressure distribution focused on the maximum contact pressure. The data conform the normal distribution and were analyzed by paired *t*-test. When the data does not conform to the normal distribution, the non-parametric test (Wilcoxon rank sum test) is used for analysis. normal distributions Descriptive statistics were calculated as the Mean ± SD.

## Results

The detailed results are in Table 3.

**Table 3:** Summary of mechanical characteristics of knee joint in during stair ascent and descent

	POPC (Mise,Mpa)	PCP(Mise,Mpa)	POFTC(Mise,Mpa)	FTCP^a^(Mise,Mpa)	Contact area^a^ (mm^2)^
Stair ascent	1.79 ± 0.07	2.82 ± 0.08	1.81 ± 0.07	3.03 ± 0.11	434.32 ± 1.70
Stair descent	1.70 ± 0.04	2.59 ± 0.06	1.59 ± 0.03	2.57 ± 0.06	249.27 ± 1.35
t/Z	-2.668	-2.666	-2.668	29.936	628.491
*P*	0.008	0.008	0.008	0.00	0.00

Abbreviations: *POPC* The pressure of patellar cartilage, *PCP* Patellar contact pressure, *POFTC* The pressure of femoral trochlea cartilage, *FTCP* Femoral trochlear contact pressure

^a^Paired *t*-test

### Magnitude of the force

During the stair descent (knee flexion at 30°), the pressure of patellar cartilage (Von Mises stress, the resultant force of normal stress and shear stress) of the patellar cartilage was 1.70 ± 0.04Mpa, the contact pressure of the patella cartilage was 2.59 ± 0.06Mpa, the pressure of femoral trochlea cartilage was 1.59 ± 0.03Mpa, the contact pressure of the patella cartilage was 2.57 ± 0.06Mpa.

During the stair ascent (knee flexion at 60°), the pressure of patellar cartilage was 1.79 ± 0.07 Mpa, the contact pressure of patellar cartilage was 2.82 ± 0.08Mpa. The pressure of femoral trochlea cartilage was 1.81 ± 0.07 Mpa, the contact pressure of femoral trochlea cartilage was 3.03 ± 0.11Mpa.

### Characteristics of the contact area and position

The contact area between patellar cartilage and femoral trochlea cartilage was 249.27 ± 1.35mm^2^ during the stair descent, which was less than 434.32 ± 1.70mm^2^ during the stair ascent. Not only the total contact area but also the area of high stress was larger during the ascend stairs.

**Fig. 7:**
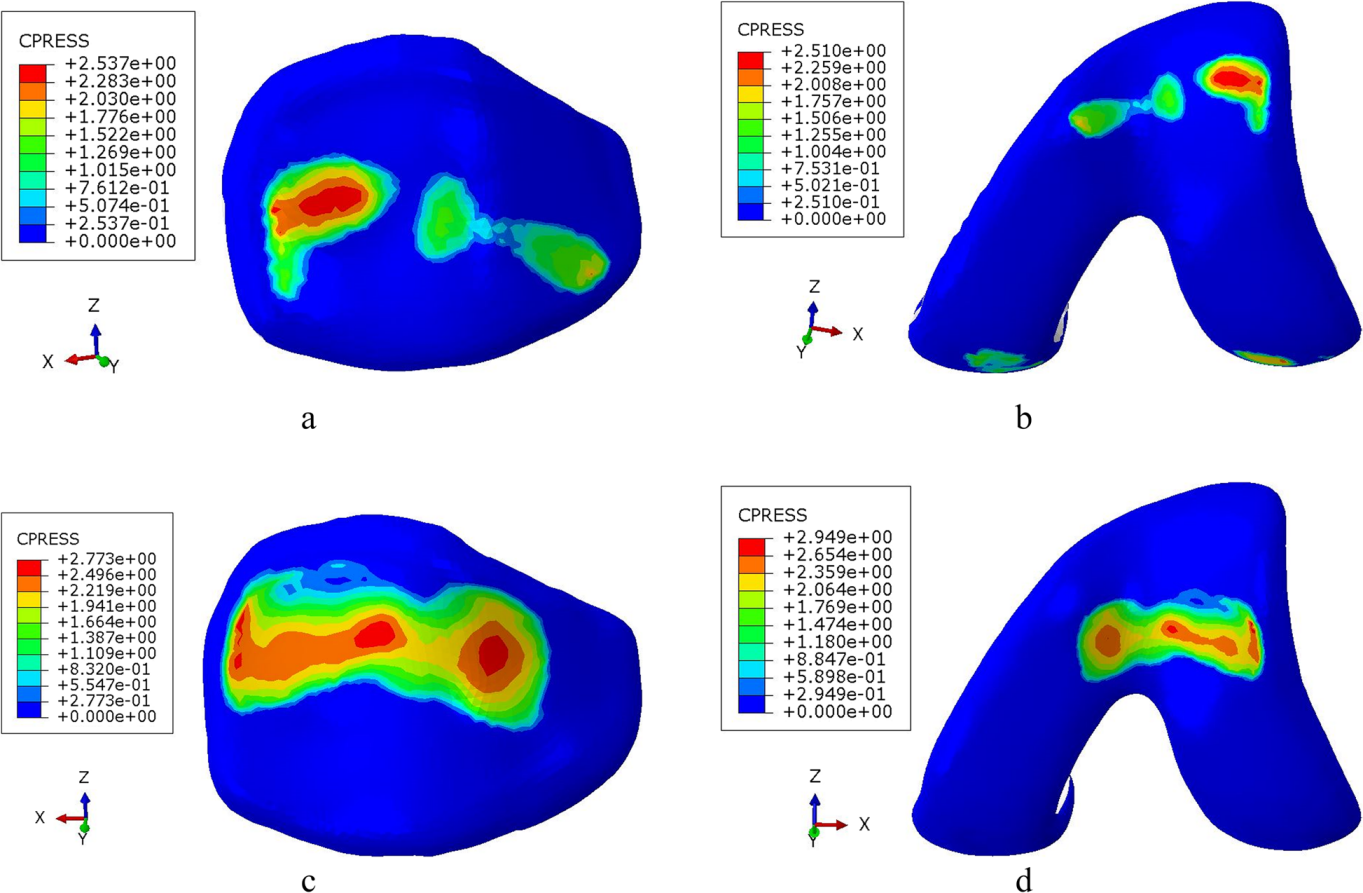
Pressure on patellofemoral articular cartilage. **a**: The contact pressure of patellar cartilage during the stair descent. **b**: The contact pressure of femoral trochlea cartilage during the stair descent. **c**: The contact pressure of patellar cartilage during the stair ascent. **d**: The contact pressure of femoral trochlea cartilage during the stair ascent

**Fig. 8:**
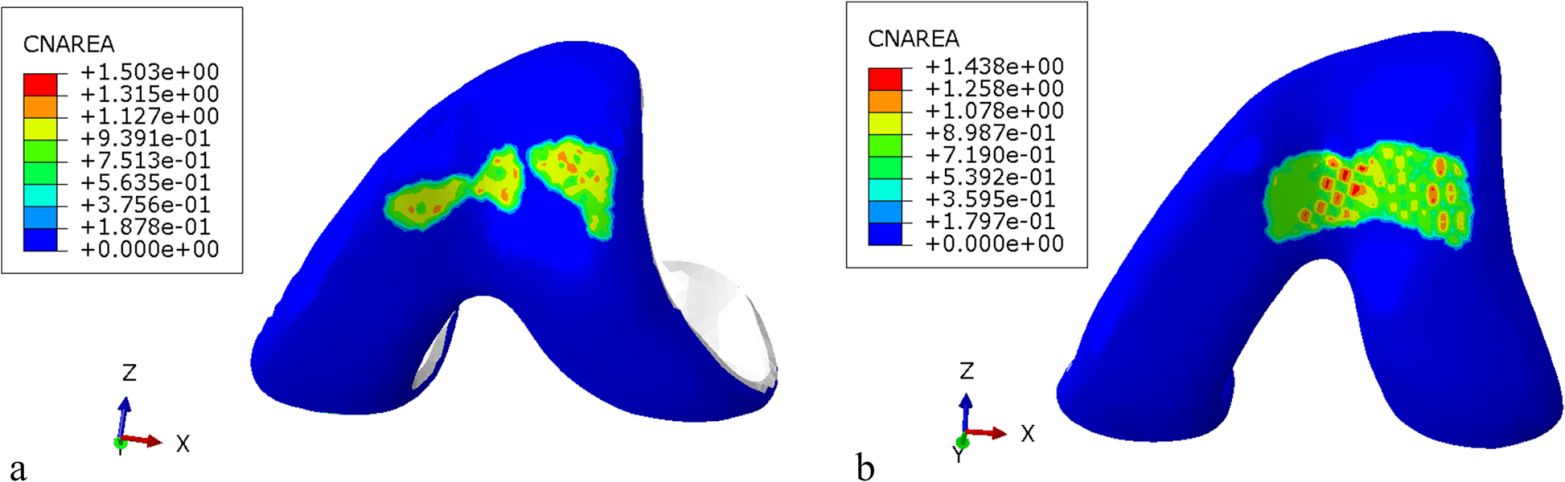
Pressure distribution characteristics of patellofemoral articular cartilage. **a**: The feature of patellofemoral articular cartilage contact area during the stair descent. **b**: The feature of patellofemoral articular cartilage contact area during the stair ascent

The contact position was located in the middle and lower part of the patella. The left side of this part was the high stress area during ascend stairs, demonstrating the force was relatively unbalanced. The contact position of patellofemoral joint was the middle and upper part of the patella during descent stairs, and the high stress area was scattered, which was relatively balance at this state. Pressure distribution characteristics of patellofemoral articular cartilage were shown in the Figs. 7  and 8.

## Discussion

In this study, a finite element knee model with 3D was developed, it provided stresses and contact areas, thus allowing for more systematic biomechanical evaluation of the patellofemoral joint during stair ascent and stair descent. This study confirms that the patellofemoral joint contact area was greater during stair ascent than that during stair descent. The patellofemoral cartilage pressure increases but fluctuates less.

Wallace et al. [22] demonstrated that patellofemoral stress increased from 30° to 90°, peaking at 90° for muscle contractions. However, more recent study has shown that peak patellofemoral contact pressures are also observed at lower knee flexion angles [23–25]. It is proved, indicating the peak knee moment is reported to occur at about 60° during stair climbing [16]. Huang [26] hold the opinion that patellofemoral joint showed the highest peak contact pressure at 1.2 MPa at the initial flexion and the patellofemoral joint pressure increases slightly at the knee flexion reaches 60°. This result was also verified in this study, the patellofemoral pressure was greater during the stair ascent (60°) than that during stair descent(30°), it has increased 0.23Mpa.

The stair ascent is sometimes noted to be more demanding on the knee than descent stair. However, some scholars believe that the two activities are quite similar in terms of reaction load in the patellofemoral joint [15]. There is little difference in the contact pressure of patellofemoral cartilage between stair ascent and stair descent in this study. Why does the patellofemoral pressure increase with the knee flexion angle increase, but the maximum pressure difference of the patellofemoral joint increase less? With the knee flexes, the patella becomes engaged within the trochlear groove and contact area increases [27]. Using cadaver limbs and pressure sensitive film, Powers [25] reported a 68% increase in contact area between 15° and 60° knee flexion, whereas D’Agata [28]reported an 81% increase between 20° and 60°. Between knee flexion 20° and 60°, an 80% increase in total contact area was observed in the Gretchen’s research [29]. An 74% increase in total contact area was observed in our research with knee flexion from 30° to 60°. We believe that the reason for the small change in pressure may be the increase in the contact area as to the findings of other scholars [27, 30, 31]. The patellofemoral joint reduces the contact pressure of articular cartilage by increasing the contact area, which is a protective mechanism for the joint.

Akbarshahi [32] measured patellofemoral pressure in 4 healthy individuals during stair ascent and they found that the contact force and stress were greater on the lateral patellar facet compared with the medial facet. The peak patellofemoral contact pressure ranged from 3.7 to 6.1 times body weight on the lateral facet compared with 0.7 to 1.3 times body weight on the medial facet [32]. Other scholars have also identified that the distribution of pressure across the articular surface was remarkably uniform [13, 33]. In the knee flexion of 30°, with the patella engaged in the trochlea, the contact pressure on the lateral patellofemoral joint surface increased in this study. But, the medial–lateral patellofemoral joint compartment of the force during the 60° of knee flexion did not differ. Our results are similar to those of the cadaver specimens conducted by Huang et al. [26]. It can be considered that the change of pressure distribution is related to the characteristics of patellar tracking during knee flexion. Some studies revealed that the patella inclined medially first and then laterally during the extension to flexion [34]. Wilson [35] believed the patella inclined medially to -1.8° within the 0°-45° range, and inclined laterally to 2.5° within the 45°-90° range.

The discrepancies between these studies are most likely related to inherent differences in study design, such as detection methods or force simulation methods [2, 24–26]. There are some limitations in the current study. (1) The reconstruction and registration of bone models can induce errors. The errors could be minimized by improving the reconstruction and registration algorithm. (2) Only quadriceps loading was applied in the anatomical directions. (3) The effects of individual anatomical differences did not investigate. These limitations might have influenced our outcomes.

## Conclusion

The patellofemoral joint contact area was greater during stair ascent (60° of knee flexion) than that during stair descent (30° of knee flexion). But there is a small change in patellofemoral cartilage contact pressure. It indicates that the joint adjusts the contact pressure by increasing the contact area.

### Supplementary Information

**Additional file 1.**

## Data Availability

All data generated during this study are included in this published article and its supplementary information files.
